# Factors That Help and Hinder the Implementation of Digital Depression Prevention Programs: School-Based Cross-sectional Study

**DOI:** 10.2196/26223

**Published:** 2021-08-27

**Authors:** Joanne R Beames, Lara Johnston, Bridianne O'Dea, Michelle Torok, Helen Christensen, Katherine M Boydell, Aliza Werner-Seidler

**Affiliations:** 1 Black Dog Institute University of New South Wales Randwick Australia

**Keywords:** secondary school, depression, prevention, digital, barrier, facilitator, teacher, counselor, principal, student

## Abstract

**Background:**

Digital prevention programs that are delivered in a school environment can inoculate young people against depression. However, little is known about the school-based factors that help and hinder the implementation of these programs. Staff members are integral for supporting mental health programs in schools and are likely to have a wealth of expertise and knowledge about the factors that affect implementation.

**Objective:**

The primary objective of this study was to explore the barriers and facilitators to implementing a digital depression prevention program in Australian secondary schools with teachers, counselors, and principals. The secondary objective was to explore variations in these factors across different school contexts, including the school type (government or nongovernment), location (capital city, regional/or rural areas), and socioeconomic status (SES) (low, medium, high).

**Methods:**

This quantitative cross-sectional survey study assessed the barriers and facilitators to implementing a hypothetical digital prevention program in Australian schools. The survey was taken by 97 teachers (average age 38.3 years), 93 counselors (average age 39.5 years), and 11 principals (average age 50.9 years) across Australia between November 2017 and July 2018.

**Results:**

A range of barriers and facilitators relating to logistics and resources, staff support, and program factors were endorsed by the surveyed staff. Consistent with prior research, common barriers included a lack of time and resources (ie, staff and rooms). These barriers were particularly evident in government, rural/regional, and low socioeconomic schools. Other barriers were specific to digital delivery, including privacy issues and a lack of clarity around staff roles and responsibilities. Facilitators included upskilling staff through training, embedding the program into the curriculum, and other program factors including universal delivery, screening of students’ mental health, and clear referral pathways. Knowledge about the program efficacy was also perceived as important by a large proportion of the respondents.

**Conclusions:**

The digital depression prevention program was perceived as suitable for use within different schools in Australia, although certain factors need to be considered to enable effective implementation. Logistics and resources, support, and program factors were identified as particularly important for school-based implementation. To maximize the effectiveness in delivering digital programs, implementation may need to be tailored to the staff roles and school types.

## Introduction

Depression is a debilitating problem in young people. An earlier onset is associated with a more severe clinical course and greater risk of recurrence, impairments in academic performance, social problems, and increased risk of comorbid physical and mental health problems [1,2]. Access to evidence-based treatment for depression is often limited, and even among those who do obtain access, relapse rates remain high [3]. Therefore, depression is a prime target for prevention. To increase access to and effectiveness of prevention efforts, a proactive approach is needed whereby programs are delivered in contexts frequented by young people using methods in which they are already engaged. One way to increase access and effectiveness is by delivering psychological prevention programs in schools.

Schools are ideally placed to address the mental health problems of young people. Young people spend much of their waking time at school and have reported a greater willingness to access mental health services at school than in other settings [4,5]. Among young people who do receive mental health care services, more than 50% have indicated that the first access was driven by their school [4]. Schools in many countries, including Australia, Canada, and the United Kingdom, typically have designated mental health and well-being staff (eg, counselors, psychologists, or well-being officers). Although there are variations across schools, these staff members are well positioned to support mental health programs and create meaningful impact for students.

School-based depression prevention programs are already available. Two meta-analyses have found that school-based prevention programs had a small preventive effect on depressive symptoms, regardless of whether the program was delivered to all students (ie, universal) or to a targeted sample [6,7]. Most of the studies involved programs that were delivered in person, an approach that places high demand on the already limited resources in schools (eg, staff availability, infrastructure, time, financial cost). Only two studies from this review reported on programs that were delivered online via a website [8,9]. More recent randomized controlled trials have investigated the effectiveness of online or telephone-based prevention programs delivered in schools, with mixed results [10-13]. Given that there is some indication of the effectiveness of school-based digital programs, using technology to deliver prevention programs may be a promising way forward in addressing student depression in schools.

### Barriers and Facilitators to Delivering Mental Health Programs in Schools

#### Digital Programs

Little is known about the factors affecting the implementation of digital mental health programs in schools. Research has typically focused on the factors that generally affect the acceptability of and engagement with digital mental health programs, or on settings other than schools [14,15]. Schools are complex environments for delivering digital mental health programs, and many factors will affect their implementation and uptake by students. Given that school staff and school leaders decide what programs are implemented, and are often involved in supporting program delivery, it is important to consider their perspectives about what will work in their schools. Research investigating the barriers and facilitators of specific digital mental health programs as perceived by school staff is currently lacking [16], with more focus on face-to-face delivery methods.

#### Face-to-Face Programs

Studies have identified school-specific factors that affect implementation of face-to-face evidence-based mental health programs. One early qualitative study involved interviews with the developers of multiple evidence-based school mental health interventions [17]. Results identified the following seven factors as being important for effective implementation: (1) principal and other administrative support; (2) teacher support; (3) adequate financial resources; (4) high-quality training and consultation to ensure fidelity to the intervention components; (5) alignment of the intervention with the philosophy, culture, and approach to mental health of the schools; (6) visibility of the intervention outcomes to key stakeholders; and (7) adequate ways to address turnover of the staff involved in program support and delivery. Another study was conducted based on these results by incorporating the perspectives of program directors and clinicians working within schools [18]. In this study, Langley and colleagues found that the four main barriers to implementation included competing responsibilities, parent engagement, logistics, and support from administrators and teachers. Facilitators included having a professional network and being able to consult others about the program, adequate funding, and perceptions that the program would be easy to implement. Other survey studies involving school staff (eg, headteachers, teachers, counselors) in middle and secondary schools have obtained converging results [19-26]. School Mental Health ASSIST, a provincial implementation support team in Ontario that aims to build school capacity to use evidence-based strategies and services, has also identified a “top 10 list” of factors that influence implementation of student mental well-being practices. These factors include commitment, leadership team, assessment of the (initial) capacity and resources, and professional learning [27]. This literature underscores the need to consider factors unique to the school environment that influence the delivery of mental health programs, rather than relying on the more generic implementation literature.

### Current Study

To the best of our knowledge, no studies have evaluated the barriers and facilitators to implementing a school-based digitally delivered depression prevention program for students as perceived by school staff. Although insights can be drawn from studies evaluating the implementation of evidence-based face-to-face interventions, there are clearly unique factors to consider in the implementation of digital mental health programs.

The current study explored the perspectives of Australian secondary school staff who are primarily responsible for the adoption and delivery of such programs in the school environment. Integrating the perspectives of principals, counselors, and teachers will provide a comprehensive picture of the factors that specifically affect the implementation of digital health programs. Based on the available literature [17,18,25,26], anticipated barriers included a lack of time and curriculum constraints, limited staff to support delivery, competing responsibilities, lack of support from the leadership and other staff members, lack of knowledge or skills to facilitate delivery, and lack of perceived fit with school values; anticipated facilitators included appropriate resourcing, a clear program champion, increased school support for staff delivering the program, perceived effectiveness of the program, and knowing what implementation involves. In addition, beliefs and attitudes toward prevention and using technology to deliver mental health programs (eg, effectiveness, safety concerns, inbuilt flexibility) were expected to influence perceptions about the ease, or difficulty, of implementation. This study also explored whether factors identified by school staff act as barriers or facilitators to implementation to the same extent across different school contexts (eg, types of schools, socioeconomic status [SES]). Given the similarities in the experiences of school staff across countries (eg, high workload, burnout, role ambiguity) [28-30], the results are likely to have relevance beyond the Australian context.

## Methods

### Design and Recruitment

This study involved online surveys of secondary school staff from Australia, including principals and deputy principals, teachers (including librarians, support teaching staff, administrators, and Year Advisors), and school counselors and psychologists. The study procedures received ethical approval from the University of New South Wales Human Research Ethics Committee (HC17468) and the State Education Research Applications Process (SERAP2017339). Recruitment involved convenience and snowball sampling at key times (eg, beginning of school terms) between November 2017 and July 2018. Flyers and emails were sent to the research team’s network of school contacts throughout New South Wales, and online flyers were posted on the Black Dog Institute’s media channels (eg, Twitter, Facebook, and the Black Dog Institute website). This was an “open survey,” and eligible participants were also encouraged to distribute the research opportunity directly to others in their networks.

Interested participants clicked the online link to access the survey that matched their role and were then presented with an information statement. The statement included the names of the investigators, eligibility criteria, purpose of the study, participation requirements (eg, length and nature of the survey), reimbursement, and data storage processes. Before accessing the survey, the participants provided informed consent by ticking boxes corresponding to items such as having read and understood the information statement.

The surveys were completed between 2017 and 2018 alongside recruitment. Participants had the option of providing an email address to receive reimbursement for their time with a $20AUD electronic gift card. To ensure participant anonymity and confidentiality, email addresses were obtained using a second survey accessed via a separate URL link that was provided after completing the first survey. Responses to the second survey (ie, email addresses) could not be linked to the participants’ responses in the main survey.

### Development and Pretesting of Measures

All the three stakeholder surveys (principal, teacher, counselor) included closed and open-ended questions about their demographics, current and previous employment, and current school. Respondents were also asked to rate how responsible they thought schools should be in addressing issues related to physical health, sexual health, mental health, and substance use among students on a Likert scale as follows: 1, very responsible; 2, somewhat responsible; 3, not really responsible; and 4, not at all responsible. A hypothetical digital depression prevention program for high school students was then described to the participants to test their attitudes toward this program. The program was described as a skill-based game, drawing from cognitive behavior therapy, that students would play over 7 20-minute sessions during class time (supervised by school staff). With all the material contained in the program, participants were informed that training or specific mental health knowledge would not be necessary. The description also emphasized that the program was evidence-based and facilitated the reduction of depressive symptoms in secondary school students. A list of barriers and facilitators to the implementation of a program of this nature were then presented. Participants were asked to rate how “challenging” the potential barriers would be for introducing the program at their school on a Likert scale as follows: 1, not at all; 2, not really; 3, moderately; 4, very; and 5, extremely. Using the same scale, they were asked to indicate how “helpful or beneficial” the potential facilitators would be for introducing this program.

For each stakeholder group, the questions were tailored to ensure suitability for their role within the school. To obtain information for developing the surveys, a review of the implementation literature pertaining to evidence-based mental health programs in schools and other relevant settings was carried out. We also consulted a network of professionals within the education, mental health, and research sectors, seeking their input regarding potential barriers and facilitators to implementing mental health programs in schools. Based on all these sources of information, we generated an extensive list of potential barriers/facilitators, which we then grouped into relevant categories, such as logistics, school support, or suitability of the program. Drafts of the surveys were circulated to our network of relevant professionals for feedback, which led to further refinement of the surveys. Once the surveys were presented electronically, they were distributed to several of the research team’s contacts within each stakeholder group for testing of the usability, technical functionality, and length before recruitment for the study commenced.

### Survey Administration and Procedure

The surveys were administered via Qualtrics, an online survey platform [31]. The surveys were 20-25 pages long with approximately 5-10 questions displayed per page. All questions required a response to progress to the next page. Participants could go back to review/edit responses on earlier pages without losing information already entered. Participants were also able to save their progress and continue completing the survey during subsequent sessions. A progress bar at the bottom of each page indicated approximately how far through the survey participants were, on a visual scale from 0 to 100%. Cookies were embedded on the first page of the survey, which prevented participants from using the same device to complete the survey more than once. The surveys took approximately 30-45 minutes to complete. All the survey responses were anonymous.

### Statistical Analysis

All analyses were conducted using SPSS (version 25, IBM Corporation) or Excel (Microsoft Corporation). All the available participant data were analyzed, including incomplete surveys. The survey completion rate was calculated for each stakeholder group by dividing the number of people who completed the entire survey by the number of people who consented to participate. Completion rates differed between the 3 stakeholder groups, with 55% (6/11) of principals, 83% (77/93) of counselors, and 75% (73/97) of teachers completing the survey.

We calculated the descriptive statistics for each participant sample and cumulative frequencies of the perceived barriers and facilitators. We report the cumulative frequencies for barriers and facilitators rated as “moderate,” “very,” or “extreme.” This decision was taken to focus on the most frequently endorsed, and therefore practically relevant, barriers and facilitators from the perspective of key school stakeholders.

In an exploratory set of analyses, we conducted correlations to identify the relationship between key individual characteristics and beliefs about the helpfulness of online mental health programs in general. We also examined the relationship between individual characteristics and the willingness of counselors to try different mental health interventions developed by researchers. These questions were designed to assess perceptions about digital mental health programs in general. We conducted chi-square tests of independence, or the Fischer exact test of independence when expected numbers were <5, to determine whether the selected school variables were associated with perceived facilitators and barriers. The variables included the type of school (government or nongovernment), school location (capital city or rural/regional), and SES (low, medium, high). These variables are related to the availability of mental health staff and other resources in schools and can therefore affect implementation processes [32-34]. For all the analyses, the level of significance was set at *P<*.05 and strength of the association was represented by Cramer’s V.

## Results

### Participant Characteristics

The survey was taken by 97 teachers, 93 counselors, and 11 principals. On average, the teachers were 38.3 years old and had been in the role for 9.8 years. The school counselors were aged 39.5 years, with 6.9 years of experience in their role, and the principals were 50.9 years old, with 10 years of experience in their role. Most teachers (85/96, 89%) and counselors (62/91, 68%) were employed in government schools, whereas less than half (5/11, 46%) of the principals were from government schools. Most participants were employed fulltime (teachers: 78/97, 80%; counselors: 65/93, 70%; principals: 11/11, 100%). The perceived SES and location of employment (eg, capital city vs. rural/regional locations) varied across respondents. Table 1 presents the details.

**Table 1:** Summary of participant and school characteristics (given that incomplete and complete surveys were analyzed, the sample size for each characteristic may vary).

Characteristics				Teachers (n=97)		School counselors (n=93)		Principals (n=11)
Gender, female, n (%)				73 (75.3)		83 (89)		4 (36)
Age (years), mean (SD)				38.3 (11.0)		39.5 (11.2)		50.9 (7.9)
Aboriginal or Torres Strait Islander, n (%)				6 (6)		2 (2.2)		0 (0)
Born in Australia, (%)				89 (92)		79 (85)		9 (82)
**Marital status, n (%)**								
	Married/partnered		72 (74)		82 (88)		10 (91)	
**Educational history, n (%)**								
	Postgraduate degree		41 (42)		82 (88)		9 (82)	
**Employment status, n (%)**								
		Full time	78 (80)		65 (70)		11 (100)	
Years in role, mean (SD)				9.8 (9.0)		6.9 (6.5)		10.05 (12.8)
**School type and location, n (%)**								
	Government schools		85 (89)^a^		62 (68)^c^		5 (46)	
	Coeducational		91 (95)^a^		75 (82)^c^		10 (91)	
	Low socioeconomic status		40 (42)^a^		33 (36)^c^		3 (27)	
	Located in a capital city		23 (24)^a^		56 (62)^c^		8 (72)	
	Located in a rural/regional town		73 (76)^a^		35 (39)^c^		3 (27)	
	Low socioeconomic status		40 (42)^b^		33 (36)^c^		3 (27)	
School size, mean (SD)				767.5 (318.3)^a^		908.5 (328.9)^d^		904.2 (339.0)

^a^N=96 (for teachers).

^b^N=95 (for teachers).

^c^N=91 (for school counselors).

^d^N=89 (for school counselors).

### Perceived Role of the School in Student Mental Health

All the school principals (11/11, 100%) and almost all the teachers (94/95, 99%) and counselors (89/91, 98%) felt they were either “very” or “somewhat” responsible for addressing the mental health of students. Notably, more counselors and teachers felt responsible for student mental health as compared to the number of those who felt responsible for the physical or sexual health of students, or their drug and alcohol use.

### Implementation of a Digital Depression Prevention Program

For the teachers and counselors, barriers and facilitators were divided into logistical factors, school support factors, suitability of the program, and other miscellaneous factors. For the principals, to match their roles, logistical factors were conceptualized in terms of costs and resources.

### Perceived Barriers

Tables S1 and S2 in Multimedia Appendix 1 display the facilitators to the implementation of a digital mental health program in schools perceived by teachers, counselors, and principals.

#### Logistical Factors (Including Costs and Resources)

Time was the most frequently reported barrier to program implementation by the surveyed staff, with 77% (70/91) of the teachers, 94% (84/89) of the counselors, and 64% (7/11) of the principals indicating that finding time in the school schedule would be a significant barrier. Frequencies indicated that a greater number of the surveyed counselors relative to the surveyed teachers reported that time, staff, and room availabilities were barriers to implementation. Less than half of the principals (5/11, 45%) indicated that funding for the program would be a major barrier.

#### School Support

Less than half of the surveyed staff members felt that obtaining school support would be a significant barrier to implementation. However, there were two exceptions for the counselors, who rated obtaining administrative support (54/89, 61%) and teacher support (53/89, 60%) as at least “moderate” challenges.

#### Program Suitability

Very few members of the surveyed staff were concerned that the program would increase the risk of mental illness (15% [13/88] of the teachers and 7% [6/87] of the counselors), and fewer than one-third of the teachers and counselors felt that mental health interventions should be conducted face to face rather than online. In comparison, 36% (4/11) of the principals indicated concern about the delivery format. Less than 11% of the teachers, counselors and principals felt that mental health programs were only appropriate to those with symptoms, suggesting acceptance of universal preventive approaches. A noteworthy proportion of the teachers (28/88, 32%) and counselors (40/87, 46%) expressed concerns that student privacy would be a significant barrier to the implementation of the digital program.

#### Other Factors

Student engagement was reported by more than half of the teachers and counselors as a potentially significant barrier to implementation. Only a small number of teachers felt that delivering this program was not their role (20/87, 23%), but many more (39/87, 45%) expressed concerns that the program would uncover mental health problems that they were not equipped to address. In contrast to the teachers, over a third of the surveyed counselors reported that delivering the program was not their role (32/87, 37%). A similar proportion of the counselors (28/87, 32%) indicated that dealing with student mental health issues and high-risk students identified during the program would be a significant barrier. Compatibility between the program and school values was also identified as a barrier by 27% (3/11) of the surveyed principals.

### Perceived Facilitators

Tables S3 and S4 in Multimedia Appendix 1 display the facilitators to the implementation of a digital mental health program in schools perceived by teachers, counselors, and principals.

#### Logistical Factors (Including Costs and Resources)

Just over 80% of the surveyed teachers (72/86) and counselors (73/87) reported that allowing students to use personal devices to access the mental health prevention program would be helpful or beneficial to implementation. Having the program available at no cost was also rated as an important facilitator by all surveyed teachers (82/82, 100%) and most counselors (83/84, 99%), but only by 70% (7/10) of the principals.

#### School Support

The surveyed teachers and counselors indicated that all aspects of school support were important facilitators to program implementation. Although results were generally comparable between the teachers and counselors, having support from the principal (83/87, 95%), teachers (84/87, 97%), administrative staff (83/86, 97%), and parents (83/86, 97%) was particularly important for counselors, as was having a staff member responsible for answering concerns (83/86, 97%) and sharing responsibility for implementation with other staff members (84/86, 98%). In contrast, the surveyed principals indicated that support from counselors/well-being staff was most important (9/10, 90%).

#### Program Suitability

All or almost all the surveyed staff indicated that knowledge on program efficacy (for academic and emotional outcomes) would be important for implementation in schools.

#### Flexibility

All the surveyed principals (10/10, 100%) and most teachers (80/82, 98%) and counselors (81/84, 96%) reported that selecting an appropriate time in the school year for program delivery would be an important facilitator. Less than half of teachers (39/82, 48%) and 67% (56/84) of the counselors reported that delivering the program to only those students who might need it would be an important facilitator. Few teachers (18/82, 22%) and counselors (23/84, 27%) indicated that a targeted approach would be “very” or “extremely” important, indicating that universal approaches may be preferred.

#### Other Factors

The available training and the format of that training were rated by teachers (76/82, 93%) and counselors (77/84, 92%) as potential facilitators to delivery. Most surveyed teachers (76/82, 93%) and counselors (76/84, 90%) and all principals (10/10, 100%) indicated that having a screening component to identify students at risk, with student information being transferred to counselors, would be at least moderately helpful. Most surveyed teachers (72/82, 88%) indicated that aligning the program with the existing school curriculum was important, and this was comparable to the ratings provided by approximately 90% (76/84) of the counselors and principals (9/90). Alignment with the school’s philosophy, knowledge on the efficacy and benefits, and receiving feedback about the impact/success of the program from students were rated as important facilitators by all the groups.

### Exploratory Analyses

Correlation analyses showed that for the surveyed teachers, age (*r*=0.03, *P*=.79) and years of experience (*r*=0.02, *P*=.88) were not associated with beliefs about how helpful online mental health programs could be in addressing common mental health problems in young people. A similar pattern of results was found for counselors (*r*=–0.06, *P*=.57 and *r*=–0.05, *P*=.67, respectively). Additionally, for counselors, there was no association between age (*r*=–0.16, *P*=.13) and years of experience (*r*=0.007, *P*=.95) with the reported willingness to try new and different types of therapy or interventions developed by researchers.

Tests of independence showed that the ratings provided for the perceived barriers and facilitators by the surveyed teachers and counselors were generally consistent across different school characteristics. However, there were some exceptions (see Tables 2-4 for descriptive percentages).

**Table 2:** Percentages for significant subgroup analyses with socioeconomic status.

Surveyed groups				Socioeconomic status				
				Low, n (%)		Average, n (%)		High, n (%)
**Teachers**								
	**Perceived barriers**							
		Time	28 (40)		34 (49)		8 (11)	
		Computer availability	22 (43)		26 (51)		3 (6)	
**Counselors**								
	**Perceived barriers**							
		Obtaining administrative support	19 (35)		22 (41)		13 (24)	
	**Perceived facilitators**							
		Having support from the school principal	32 (39)		27 (33)		24 (29)	
		Having the delivery of the program recognized by the principal as part of the job	28 (38)		25 (34)		20 (27)	
		Sharing responsibility for implementing the program with other staff members	31 (37)		29 (35)		24 (29)	

**Table 3:** Percentages for significant subgroup analyses with school location.

Surveyed groups					School location			
				Capital city, n (%)			Rural/regional areas, n (%)	
**Teachers**								
	**Perceived barriers**							
		Computer availability	14 (28)			37 (73)		
		Technical infrastructure and support	8 (28)			21 (72)		
**Counselors**								
	**Perceived facilitators**							
		Having support from other teachers	50 (60)			34 (41)		

**Table 4:** Percentages for significant subgroup analyses with school type.

Surveyed groups				School type		
			Government, n (%)		Nongovernment, n (%)	
**Teachers**						
	**Perceived barriers**					
		Obtaining support from other teachers	68 (87.2)		2 (12.8)	
**Counselors**						
	**Perceived facilitators**					
		Having the program aligned to the PDHPE^a^ curriculum	53 (70)		23 (30)	
		Allowing students to use of personal devices for the program	47 (64)		26 (36)	
		Having the program available at no cost	66 (55)		34 (28)	
		Sharing responsibility for implementing the program with other staff members	57 (68)		27 (32)	
		Having a training manual to support implementation	55 (68)		26 (32)	

^a^PDHPE: personal development, health, and physical education.

#### Teachers

For teachers, logistic and school support varied by subgroup. SES was significantly associated with teachers’ perceptions that finding time in the school curriculum (*P=*.001, ν=0.33) and computer availability (*P=*.004, ν=0.37) would be barriers. Analyses of the percentages showed that time and computer availability were perceived as more important by teachers from low and average SES schools compared to high SES schools. The school location was significantly associated with teachers’ perceptions that computer availability (*P=*.001, ν=0.55) and access to technical infrastructure (*P=*.02, ν=0.52) would be barriers. These factors were more likely to be barriers for teachers from rural/regional schools compared to schools in capital cities. The type of school was significantly associated with teachers’ perceptions that obtaining support from other teachers would be a barrier (*P=*.01, ν=0.56), with teacher support being as more important in government schools than in nongovernment schools.

#### Counselors

The school SES was significantly associated with the counselors’ perceptions that obtaining support from school administrations would be a barrier (*P=*.02, ν=0.31). Inspection of the percentages indicated that administrative support was perceived as a greater barrier by counselors from low and average SES schools compared to those in high SES schools.

In terms of facilitators, the school SES was significantly associated with the counselors’ perceptions that obtaining principal support (*P=*.01, *ν*=0.28), recognition from the principal that program delivery was part of their job (*P=*.04, *ν*=0.25), and sharing responsibility for introducing the program with other staff (*P=*.04, *ν*=0.26) would be important facilitators. These factors were perceived as more important facilitators by counselors from low and average SES schools compared to those in high SES schools. The type of school was significantly associated with counselors’ perceptions that aligning the program to the school curriculum (*χ*^2^_2_=6.27 [N=76]; *P=*.046; *ν*=0.29) and being able to complete the program on the students’ own devices (*χ*^2^_2_=7.57 [N=73]; *P=*.02; *ν*=0.32) would be important facilitators. For both facilitators, there was a greater importance placed on these factors by counselors in government schools compared to those in nongovernment schools. The same pattern of results was also found for counselors’ perceptions about the program being accessible for free, being provided with a training manual, and sharing responsibility for introducing the program, with *Ps*<.04 and *νs>*.29. Finally, the school location was significantly associated with counselors’ perceptions that having support from other teachers would be an important facilitator, with *P=*.02 and *ν*=0.30, with teacher support being rated as a more important facilitator by counselors from schools in capital cities than those in rural/regional schools.

## Discussion

### Summary of Findings

The current study identified barriers and facilitators that must be considered when implementing a digital depression prevention program within Australian schools. The results align with the emergence of conceptual frameworks that outline the influential factors in the implementation of school-based interventions [35]. The results also replicate and extend findings from studies investigating the implementation and use of digital mental health programs in young populations [14,36], as well as mental health programs delivered in schools via traditional methods (eg, face-to-face programs) [17,25,37]. We clarify our results in the context of this prior research in the sections below.

### Barriers to Implementation

Overall, teachers, counselors, and principals thought that a digital depression prevention program would be suitable for use within their schools, although certain factors would need to be considered to enable effective implementation. Lack of time and resources (ie, staffing and rooms) were identified as logistical barriers. This is consistent with prior work investigating the barriers for implementing face-to-face mental health programs in schools [17,25,37] and is not particularly surprising given the high workload and demands of school staff. Although endorsed by all the surveyed staff, a novel finding in our study was that these logistical barriers were particularly evident for counselors. Counselors are expected to be more familiar with what is needed to implement mental health programs effectively in schools owing to their experience in their role. Identifying differences in terms of the degree rather than the kind is an important addition to literature, with the implication being that some strategies to boost implementation ought to be tailored to specific staff members.

Teachers and counselors are concerned about information privacy in school-based digital mental health programs. Common concerns about privacy in digital approaches include how personal information about students will be stored, accessed, and used [38]. Some counselors were also concerned about having to address student mental health issues identified through the program (particularly those that are highly risky). These perceived barriers might reflect the uncertainty about how counselors will be ethically informed about students’ needs and how they will provide effective care. These issues of privacy require novel solutions that are not currently addressed in face-to-face mental health care programs.

Another result supplementing the current literature was that over a third of the counselors thought that delivering the program was not their role. One explanation is that these counselors had reservations about delivering programs via digital means, and perhaps felt unequipped in terms of technical knowledge. This is an unlikely reason because most counselors (indeed, most respondents) indicated that a digital format was appropriate for students in their schools. Another possible explanation is that because the digital program would deliver standardized mental health content, counselors recognized that delivery would require low levels of support and mental health expertise, which could potentially be delegated to other school staff members such as teachers. This would benefit them in that they can have more time to work with higher-risk students and provide follow-up care. This interpretation converges with our finding that not having support from other administrative staff and teachers would be a major barrier to implementation for counselors. However, some teachers were concerned about having to help students with problems that they were not equipped to address. These results build on previous findings by indicating that new ways of collaborating with other staff might be necessary for successful implementation. Clear implementation guidelines need to be established before digital prevention programs are implemented in schools.

### Facilitators to Implementation

Specific to digital programs, teachers and counselors thought that using personal devices was an important facilitator. Allowing students to use their own devices may overcome limitations in terms of school infrastructure/resources. Teachers and counselors also stated that receiving training and a training manual would assist with implementation. Training could increase staff confidence and buy-in to the program by clarifying the rationale and evidence for implementing the program, staff responsibilities, and prerequisite mental health knowledge. Previous research has shown that mental health literacy is an unmet need in school staff, with many wanting additional training to support their professional development [39-41]. Further, we discovered evidence for embedding digital programs into the national curriculum, with many school staff members indicating that existing school subjects (such as health and physical education) could be used as the timeslots for delivery. Integration into the curriculum would not only save time but also help consolidate support from the leadership (education departments, principals) and other staff within schools (eg, administration). Finally, the principals indicated that support from the counselor or well-being staff was the most important facilitator for successful implementation. Having support from staff with mental health expertise will help ensure that suitable programs are selected and that benefits are maximized for students.

Specific characteristics of the program were also identified by the surveyed staff as important facilitators. These characteristics included having an evidence-based, universal delivery, inclusion of a screening component, and clear referral pathways. A large proportion of the teachers, counselors, and principals indicated that knowledge on the program’s efficacy would be important for implementation. Very few evidence-based mental health programs are implemented in schools; however, when used, they are not typically implemented as intended [34,42]. Respondents also thought that universal delivery of the program to all students, regardless of their symptoms, would increase the ease of implementation. This approach aligns with meta-analytic data showing that universal prevention programs, including those delivered using technology, are effective when delivered in schools [7].

The staff reported that incorporating a screening component to identify students at risk, with student information then being transferred to a counselor, would be necessary in the school context. Mental health screening in schools has the advantage of identifying at-risk students early and preventing them from slipping through the cracks [43-45]. In turn, engaging a triaging system would help to allocate school resources based on student needs. Previous research in Australian schools has shown that using an online mental health screening service in schools is an effective way of identifying students in need and providing an appropriate level of care [46].

### School-Based Factors

There were variations in the perceived implementation barriers and facilitators based on school contextual factors. Similar to previous research [33], the school location, type, and SES played a role in shaping the teachers’ and counselors’ perceptions regarding which factors would influence the implementation success. The general pattern was that the staff working in government schools, rural/regional areas, and low socioeconomic catchments anticipated facing more logistical and support barriers during implementation. These staff members identified a complementary set of facilitators to maximize implementation in these schools. Emphasis was placed on obtaining support from the other staff members and leadership, sharing responsibility for the program, embedding the program into the personal development, health, and physical education (PDHPE) curriculum, providing the program at no cost, and allowing students to use their own devices. These facilitators can be viewed as ways to reduce the burden on the already limited resources and fit the program within schools.

### Implications

**Figure 1:**
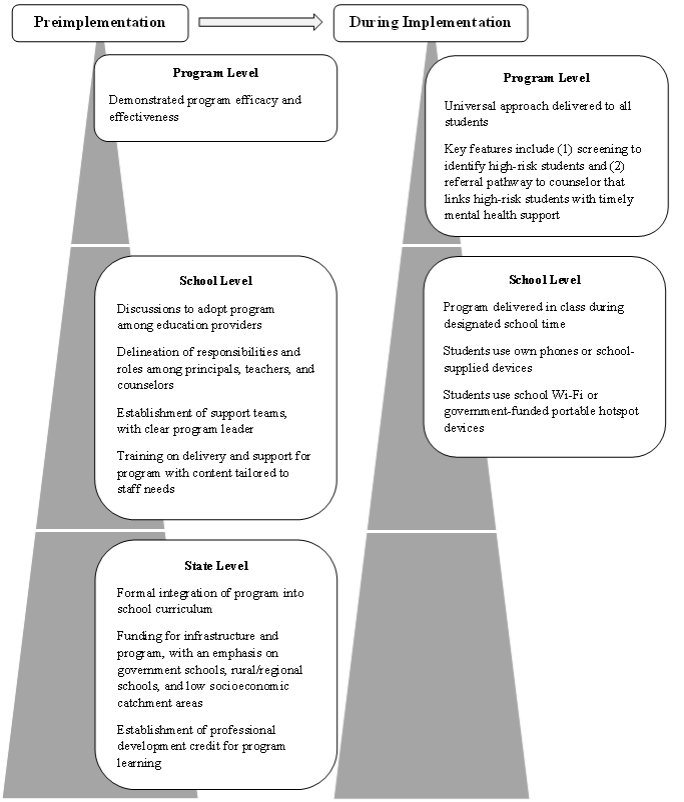
Key implementation factors to consider in digital mental health program delivery at the program, school, and state levels, with focus on the Australian context.

Our results indicate that different approaches are needed to target various staff members in different school types. One example is involving counselors in discussions about the program, before implementation, to ensure that they are on-board and can provide support. This might be particularly important in government schools. Another example is tailoring program implementation strategies to school regions and ensuring preparedness in terms of available technology, infrastructure, and support [32]. Our results also highlight the importance of factors that impact decisions related to adopting digital programs ahead of time [47]. Some face-to-face programs are already being developed with school-based barriers and facilitators in mind [48]. Extending this research to digitally delivered programs may help to design and develop school appropriate products. Figure 1 shows the proposed outline of the key implementation factors formulated from the current results.

### Limitations

This study is limited in some respects. The sampling processes could have contributed to selection biases and positive self-presentation. School staff who responded to the survey may have already been interested or engaged in the mental health of their students. Staff members who were reached by recruitment but did not consent to participate may have had systematically different perspectives about the anticipated barriers and facilitators. Further, our study only included a small proportion of the total number of staff members in Australia eligible to participate in the study. However, the teachers, counselors, and principals in our study did represent a variety of school types, locations, and socioeconomic circumstances, which increases the generalizability of our findings. The characteristics of our sample, including the average age and gender proportion, were also consistent with the population characteristics of Australian teachers and principals [49]. Random sampling with more participants will help to prevent biases in sampling during future studies.

Another limitation is that we only focused on schools in Australia. Although our results may be relevant in other Australian states and countries with similar school systems (eg, United Kingdom), it is unclear how digital programs would be received in places with fewer resources or different staffing roles. Future research could compare the implementation factors in schools on an international level, with particular focus on schools in low- and middle-income countries (LMICs). LMICs are likely to encounter different barriers and facilitators, issues that are important to consider when implementing such programs on a large scale [50]. Finally, given that we examined a hypothetical digital depression prevention program, our conclusions may not be generalizable to the actual implementation of real-world programs. Implementation process evaluations of digital mental health programs will help understand what the most effective approach in schools is, thereby improving outcomes for students and school communities. One example of such an evaluation is currently underway in Australian schools [16,51].

### Conclusions

Our study explored the factors influencing the implementation of a digital depression prevention program within secondary schools from the perspectives of teachers, counselors, and principals. The surveyed staff members thought that a digital program focusing on universal prevention was suitable for delivery in their schools. A range of logistical, support, and program barriers and facilitators were identified, some of which were unique to digital programs. There were also some differences between what teachers, counselors, and principals thought were the most important factors to be considered, as well as among staff members from different schools. Our results highlight the importance of considering multiple perspectives in school-based implementation and tailoring strategies to maximize digital delivery based on staff roles and school types.

## Appendix

Multimedia Appendix 1 Result tables for barriers and facilitators.

## Abbreviations

LMIC: low- and middle-income country
PDHPE: personal development, health, and physical education
SES: socioeconomic status
