# Supplementary material for: Factors That Help and Hinder the Implementation of Digital Depression Prevention Programs: School-Based Cross-sectional Study
Source: J Med Internet Res. 2021 Aug 27;23(8):e26223. doi: 10.2196/26223 (PMC8433863; doi:10.2196/26223)
Supplement: Multimedia Appendix 1 [file jmir_v23i8e26223_app1.pdf]

Appendix 1

Factors That Help and Hinder the Implementation of Digital Depression Prevention Programs: School-Based Cross-Sectional Study

Corresponding Author

Dr Joanne R Beames, Black Dog Institute, University of New South Wales, Sydney, NSW, Australia. j.beames@blackdog.org.au; +61 2 9382 6776.

Tables

Table S1. Barriers to the implementation of a digital mental health program in schools perceived by teachers and counselors.

Barriers	Teachers		School Counselors	
	At least moderately challenging[a], n (%)	At least very challenging[b], n (%)	At least moderately challenging, n (%)	At least very challenging, n (%)
**Logistical factors**	*N*=91		*N*=89	
Time	70 (77)	25 (28)	84 (94)	45 (51)
Staff availability	49 (54)	14 (15)	56 (63)	30 (34)
Room availability	37 (41)	13 (14)	54 (61)	27 (30)
Computer availability	51 (56)	23 (25)	43 (48)	22 (25)
Technical infrastructure and support	29 (32)	11 (12)	29 (33)	8 (9)
**School support**	*N*=90		*N*=89	
Obtaining school principal support	27 (30)	5 (6)	39 (44)	11 (12)
Obtaining support from other teachers	42 (47)	7 (8)	53 (60)	5 (6)
Obtaining administrative support	44 (49)	10 (11)	54 (61)	10 (11)
Obtaining parental support	36 (40)	5 (6)	44 (49)	8 (9)
**Suitability of the program**	*N*=88		*N*=87	
Concern the program will increase risk of mental illness	13 (15)	4 (5)	6 (7)	1 (1)
Concern therapy should be face-to-face not online	26 (30)	5 (6)	18 (21)	4 (5)
Concern mental health programs should only be delivered to those with symptoms	9 (10)	9 (10)	7 (8)	1 (1)
Concern about student privacy	28 (32)	6 (7)	40 (46)	6 (7)
**Other factors**	*N*=87		*N*=87	
Student engagement	51 (59)	12 (14)	52 (60)	13 (15)
Feeling that delivering this program is not my role	20 (23)	7 (8)	32 (37)	11 (13)
Worry that the program will uncover mental health issues I am not equipped to deal with (teachers only)	39 (45)	9 (10)	NA	NA
Responding to high-risk students uncovered by the program (counselors only)	NA	NA	28 (32)	28 (32)

[a]Data are n (%) of respondents who indicated the factor would be ‘extremely’, ‘very’ or ‘moderately’ challenging to implementation. [b]Data in brackets reflects ‘extremely’ or ‘very’.

Table S2. Barriers to the implementation of a digital mental health program in schools perceived by principals.

**Barriers**	Principals<br>*n*=11	
	At least moderately challenging[a], n (%)	At least very challenging[b], n (%)
**Costs and resourcing**		
Time	7 (64)	4 (36)
Staff availability	6 (55)	1 (9)
Funding	5 (45)	2 (18)
**School support**		
Obtaining school board support	1 (9)	0 (0)
Obtaining support from teachers	3 (27)	0 (0)
Obtaining support from counselling/wellbeing staff	1 (9)	0 (0)
Obtaining support from parents	3 (27)	0 (0)
**Suitability of the program**		
Concern the program will increase risk of mental illness	0 (0)	0 (0)
Concern therapy should be face-to-face not online	4 (36)	2 (18)
Concern mental health programs should only be delivered to those with symptoms	1 (9)	0 (0)
**Other factors**		
Ensuring the program is compatible with school values	3 (27)	2 (18)
Overcoming concern that school performance indicators are linked to academic and not emotional outcomes	2 (18)	1 (9)

[a]Data are n (%) of respondents who indicated the factor would be ‘extremely’, ‘very’ or ‘moderately’ challenging to implementation. [b]Data in brackets reflects ‘extremely’ or ‘very’.

Table S3. Facilitators to the implementation of a digital mental health program in schools perceived by teachers and counselor.

Facilitators		Teachers		School Counselors	
		At least moderately challenging[a], n (%)	At least very challenging[b], n (%)	At least moderately challenging, n (%)	At least very challenging, n (%)
**Logistical Factors**		*N*=86		*N*=87	
	Allowing students to use of personal devices for the program	72 (84)	50 (58)	73 (84)	48 (55)
**School Support**		*N*=86		*N*=87	
	Having support from the school principal	76 (88)	67(78)	83 (95)	75 (86)
	Having the delivery of the program recognized by the principal as part of the job	72 (84)	56 (65)	73 (84)	57 (66)
	Having support from other teachers	78 (91)	63 (73)	84 (97)	72 (83)
				N=86	
	Having support from parents	78 (91)	63 (73)	83 (97)	65 (76)
		N=83			
	Having administrative support	74 (89)	49 (59)	83 (97)	68 (79)
	Receiving practical support from the program development team to assist with implementation if needed	77 (93)	63 (76)	81 (94)	66 (77)
	Having a school staff member responsible for answering questions or concerns	77 (93)	65 (78)	83 (97)	69 (80)
	Sharing responsibility for implementing the program with other staff members	77 (93)	58 (70)	84 (98)	69 (80)
**Suitability of the Program**		*N*=83		*N*=85	
	Knowledge of the program's efficacy	80 (96)	63 (76)	83 (98)	77 (91)
	Knowledge that the program is beneficial for both academic and emotional outcomes	80 (96)	66 (80)	84 (99)	78 (92)
**Flexibility**		*N*=82		*N*=84	
	Deciding which age group to deliver the program to	73 (89)	49 (60)	78 (93)	57 (68)
	Deciding when in the school year to deliver the program	80 (98)	69 (84)	81 (96)	63 (75)
	Delivering the program only to those you feel need it	39 (48)	18 (22)	56 (67)	23 (27)
**Other Factors**		*N*=83		*N*=86	
	Having the program aligned to the school's philosophy	75 (90)	59 (71)	73 (85)	57 (66)
		N=82		*N*=84	
	Having the program aligned to the PDHPE curriculum	72 (88)	55 (67)	76 (90)	50 (60)
	Having a screening component to identify students at risk with information transferred to school counselor	76 (93)	56 (68)	76 (90)	64 (76)
	Having the program available at no cost	82 (100)	79 (96)	83 (99)	76 (90)
	Having the opportunity to attend face-to-face training in how to deliver the program	76 (93)	61 (74)	77 (92)	67 (80)
	Having a training manual to support implementation	78 (95)	61 (74)	81 (96)	68 (81)
	Receiving feedback about the impact and success of the program from students	79 (96)	66 (81)	83 (99)	78 (93)

[a]Data are n (%) of respondents who indicated the factor would be 'Extremely', 'Very' or 'Moderately' helpful or beneficial in being able to implement the program. [b]Data in brackets reflects 'Extremely' or 'Very'.

Table S4. Facilitators to the implementation of a digital mental health program in schools perceived by principals.

**Facilitators**		Principals<br>*n*=10	
		At least moderately challenging, n (%)	At least very challenging, n (%)
**Costs and resourcing**			
	Having the program available at no cost	7 (70)	5 (50)
**School support**			
	Having support from the school board	5 (50)	3 (30)
	Having support from teachers	7 (70)	5 (50)
	Having a school staff member selected to supervise and take responsibility for the delivery of the program	7 (70)	7 (70)
	Having support from the school counselor/wellbeing staff	9 (90)	8 (80)
	Having support from parents	7 (70)	6 (60)
**Suitability of the program**			
	Knowledge of the program's efficacy	10 (100)	8 (80)
	Knowledge that the program is beneficial for both academic and emotional outcomes	10 (100)	8 (80)
**Flexibility**			
	Deciding when in the school year to deliver the program	10 (100)	10 (100)
**Other factors**			
	Having endorsement from the Department of Education	6 (60)	5 (50)
	Having endorsement from principal leadership groups	8 (80)	6 (60)
	Having the program align with school philosophy	10 (100)	10 (100)
	Having the program aligned to the PDHPE curriculum	9 (90)	6 (60)
	Having a screening component to identify students at risk with information transferred to school counselor	10 (100)	10 (100)
	Receiving feedback about the impact and success of the program from students	10 (100)	10 (100)
	Having training available to staff who are responsible for supervising the program	9 (90)	7 (70)

[a]Data are n (%) of respondents who indicated the factor would be 'Extremely', 'Very' or 'Moderately' helpful or beneficial in being able to implement the program. [b]Data in brackets reflects 'Extremely' or 'Very'.
